# Chloroform fraction of *Chaetomorpha brachygona,* a marine green alga from Indian Sundarbans inducing autophagy in cervical cancer cells in vitro

**DOI:** 10.1038/s41598-020-78592-9

**Published:** 2020-12-11

**Authors:** Indira Majumder, Subhabrata Paul, Anish Nag, Rita Kundu

**Affiliations:** 1 Department of Botany, University of Calcutta, 35, Ballygunge Circular Road, Kolkata, 700019 India; 2 School of Biotechnology, Presidency University, Canal Bank Rd, DG Block, Action Area 1D, New Town, West Bengal 700156 India; 3 Department of Life Sciences, CHRIST (Deemed To Be University), Bangalore, 560029 India

**Keywords:** Cervical cancer, Autophagy

## Abstract

Sundarbans Mangrove Ecosystem (SME) is a rich repository of bioactive natural compounds, with immense nutraceutical and therapeutic potential. Till date, the algal population of SME was not explored fully for their anticancer activities. Our aim is to explore the potential of these algal phytochemicals against the proliferation of cervical cancer cells (in vitro) and identify the mode of cell death induced in them. In the present work, the chloroform fraction of marine green alga, *Chaetomorpha brachygona* was used on SiHa cell line. The algal phytochemicals were identified by GCMS, LCMS and column chromatography and some of the identified compounds, known for significant anticancer activities, have shown strong Bcl-2 binding capacity, as analyzed through molecular docking study. The extract showed cytostatic and cytotoxic activity on SiHa cells. Absence of fragmented DNA, and presence of increased number of acidic vacuoles in the treated cells indicate nonapoptotic cell death. The mode of cell death was likely to be autophagic, as indicated by the enhanced expression of Beclin 1 and LC3BII (considered as autophagic markers) observed by Western blotting. The study indicates that, *C. brachygona* can successfully inhibit the proliferation of cervical cancer cells in vitro*.*

## Introduction

Cervical cancer is one of the deadliest cancer of women belonging to the age group of 25 to 45 years. According to Globocan 2018, cervical cancer holds fourth position in terms of occurrence and mortality^1^. The prevalence is more in countries with low HDIs (Human Development Index), India stands 129th among 189 countries in 2019 HDI ranking, has greater risk and burden of this cancer form. In India, 469.1 million women, above the age of 15, are at risk of cervical cancer, which is the 2nd leading cause of female cancer with an estimated 96,922 new cases and 60,078 deaths per year. Among the states, highest number of incidences of Squamous epithelium carcinoma (the most common form of cervical cancer) are reported from Mizoram and lowest from Kerala^2^. According to National Cancer Institute (NCI), high risk HPVs are responsible for about 70% of cervical cancer cases with 5% of all cancer death worldwide. Along with HPVs, other cofactors (high parity, use of long term hormonal contraceptives, infection with HIV, Chlamydia, immunosuppression and dietary deficiency) are also responsible for progression from cervical infection to cervical cancer cases^2^. Several vaccines (Gardasil 9, Cervarix) have been approved against HPV 16 and 18 causing cervical cancers.

Conventional treatments involve chemotherapy and or surgery, which are associated with severe side effects and expensive to some extent. Therefore, some alternate treatment is the need of this hour. In 20th Century, use of secondary metabolites from bacterial and plant origin has gained special attention worldwide. About 30% of commercial drugs used to treat diseases has been derived from plants^3^. Marine environment covers about 75% of total Earth’s surface consisting about half of total biodiversity of the world. Both marine micro and macroalgae contain various novel bioactive compounds^4^ having antimicrobial, antineoplastic and antiviral properties. Soft-bodied marine macroalgae have various types of chemicals to protect themselves from various adversities. These phytochemicals are reported to have anti-viral, anti-bacterial and anti-fungal activities and used traditionally for medicinal purposes in India, China, Japan, Korea, Ireland, Wales and other countries. Brown seaweeds were reported to have anti-inflammatory, antitumoral and immuno-stimulant activities^5^. There is a huge scope to obtain novel bioactive compounds from these unexplored marine algae. Some isolated algal compounds were under clinical trials to check their therapeutic potentiality. Indian Sundarbans is world’s largest mangrove ecosystem containing enormous marine diversity. The algal members from Sundarbans are highly unexplored for their therapeutic role in cancer till date. Earlier it was reported from our lab that, autophagic cell death was induced in HeLa cell line by methanolic extracts of *Enteromorpha* and *Rhizoclonium* from Sundarban^6^, and cytotoxicity of six algal genera including *Chaetomorpha brachygona* in Siha cell line^7^. A very recent report from Haq et al.^8^ showed phytochemical analysis along with antioxidant and anticancer activities of another species of *Chaetomorpha* collected from Arabian Gulf^8^. They showed presence of unique anticancer compounds like Dichloacetic acid, oximes and L-α-Terpinol in the algal extract, which showed cytotoxicity in MDA-MB-231 breast cancer cells with low IC50 dose but elucidation of cell death mechanism was not studied.

In this context, present study, intends to explore the bioactive phytochemicals detected in the green algal genus *Chaetomorpha brachygona*, collected from Sundarbans Mangrove Ecosystem (SME), and the mode of cell death induced by the extract in SiHa cells through some experimental results. This is the first report of autophagic potential of *C. brachygona* collected from SME on SiHa cells.

## Results

### Chemical characterization of the extract fraction

**Figure 1:**
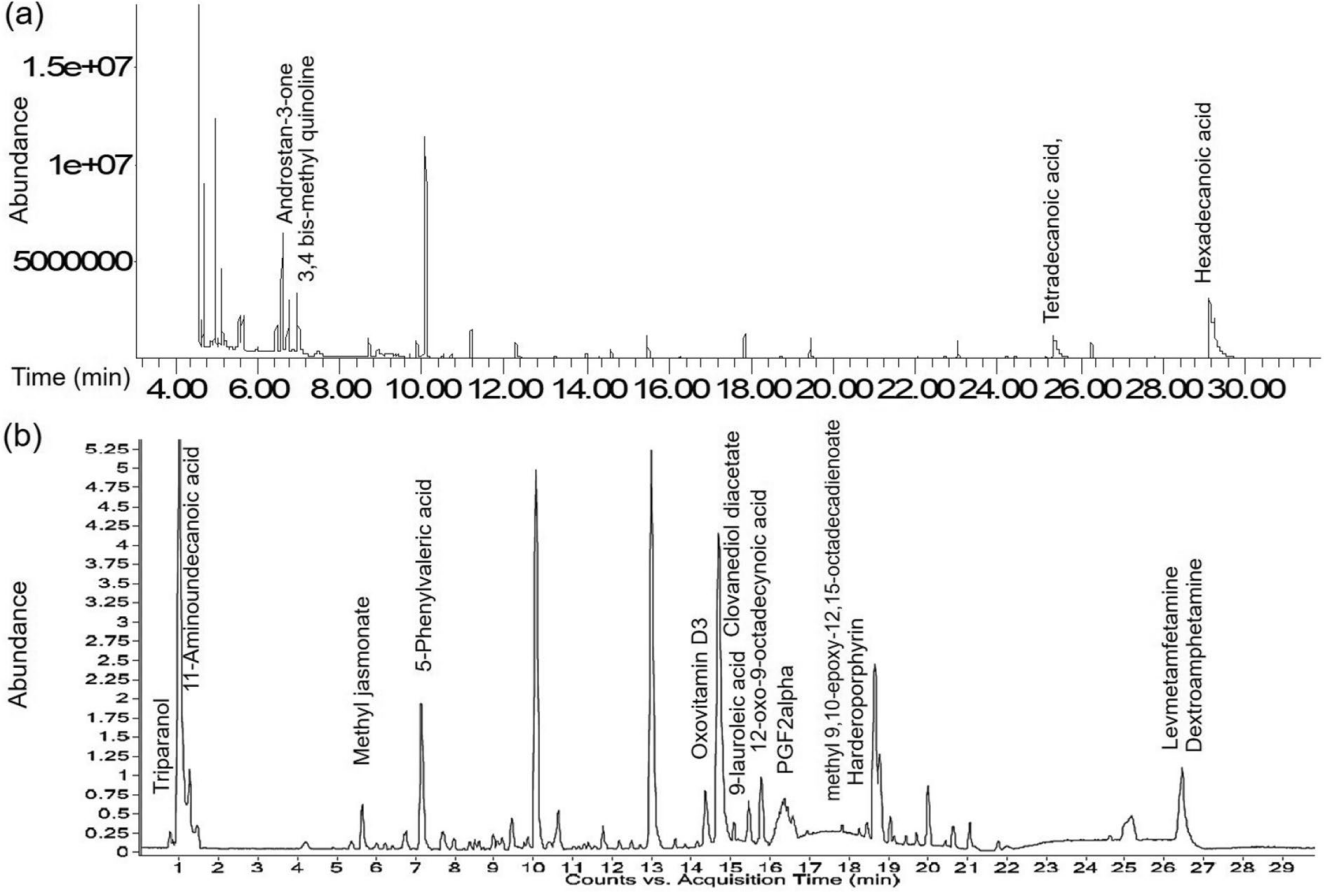
Chromatograms showing peaks of different phytochemicals present in CCF, (**a**) GC–MS (**b**) LC–MS analysis. Noteworthy phytochemicals are indicated in the chromatographs.

Several phytochemicals were detected and identified by chromatographic studies (Fig. 1). Twelve compounds were identified with GC–MS analysis. The major compounds present are- 3,4 bis-methyl quinoline, hexadecanoic acid, tetradecanoic acid and a steroid androstan-3-one (Table S1). LC–MS analysis yielded twenty-one compounds. Among those, methyl jasmonate, triparanol, undecanoic acid, phenylvaleric acid, PGF2 alpha isopropyl ester, fatty acids etc. were reported to have anti-cancer activities (Table S1).

### Anti-proliferative activity of CCF in SiHa cells

We have previously reported that, *Chaetomorpha brachygona* chloroform fraction (CCF) was found to have selective cytotoxic properties for SiHa cells, with an IC_50_ dose of 23.6 μg/ml^7^. To detect the mode of cell death in SiHa cells, studies were undertaken by treating the cells with the IC_50_ concentrations of CCF for different time points.

Five pure fractions were isolated from the extract through column chromatography. Among them three compounds were found to have no cytotoxicity (Fr. PE6, Fr. 2.17-25, Fr. 3.07) and two compounds (Fr. 3.27 and Fr. 10.01) shown cytotoxicity to SiHa cells (Fig S1, S2). Fr. 3.27 is a long chain hydrocarbon with attached Phenyl group, with a molar mass of 780. It showed an IC50 dose of 370 µg/ml. The other fraction, Fr. 10.1, was identified as 8-keto Eicosane (C_20_H_40_O) by proton NMR, with a molar mass of 298.56 g/mol, exhibited the IC50 conc. at 252.32 µg/ml (Fig S2).

**Figure 2:**
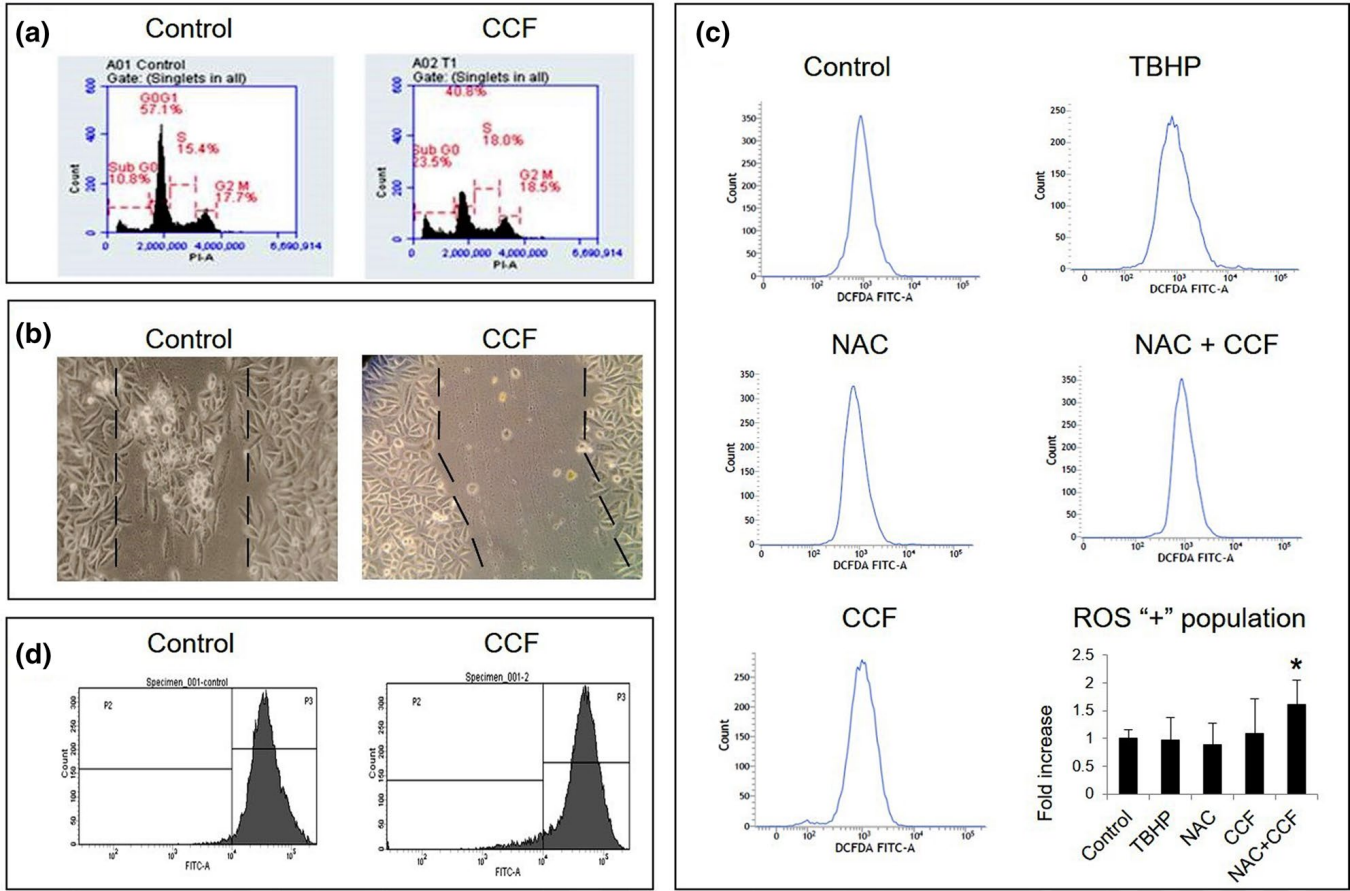
Cytotoxicity induced by CCF in SiHa cells, (**a**) Flow Cytometric analysis of Cell Cycle kinetics; (**b**) Effect of CCF on wound healing capabilities; (**c**) Flow Cytometric analysis of DCFDA stained cells for detection of intracellular ROS generation, Bar graph showing fold increase accumulation of intracellular ROS in cells, Values are expressed as mean ± SD of three independent experiments (n = 3), *denotes significant difference between control and treated sets (*P* < 0.05); (**d**) Flow Cytometric analysis of loss of mitochondrial membrane potential by Rh 123 staining.

*Cell cycle* dynamics helps us to know about the cytotoxic and cytostatic potential of a treatment. Flow cytometric analysis showed no significant change in cell cycle dynamics in CCF treated cells. The percentage of sub-G0 cells had increased about 2.5 folds while percentages of cells at S and G2-M phases were relatively same showing high toxicity of this treatment. Thus, no cytostatic activity was evident from the analysis (Fig. 2a). *Scratch/ wound healing assay* showed that, cells had regrown within the scratch marked region in control cells while, in the treated sets, the scratch marked region remained free from the migrating cells, as observed under phase contrast microscope (ZEISS) (Fig. 2b) indicating anti-proliferative activity of CCF. For intracellular *ROS generation* by CCF treatment, flow cytometric quantification of DCFDA stained cell population was found to increase significantly (about two folds) compared to the control sets (Fig. 2c). In NAC positive sets (ROS scavenger), the amount of fluorescence had decreased compared to the CCF treated set depicting no experimental error. Being a key event associated with cell death, decrease in *mitochondrial membrane potential* was prominent in CCF treated cells. Flow cytometric quantification showed two-fold increase in depolarized cell population (Fig. 2d). Increased ROS generation and decrease in mitochondrial membrane potential indicate mitochondrial stress in the treated cells.

### Elucidation of cell death type

**Figure 3:**
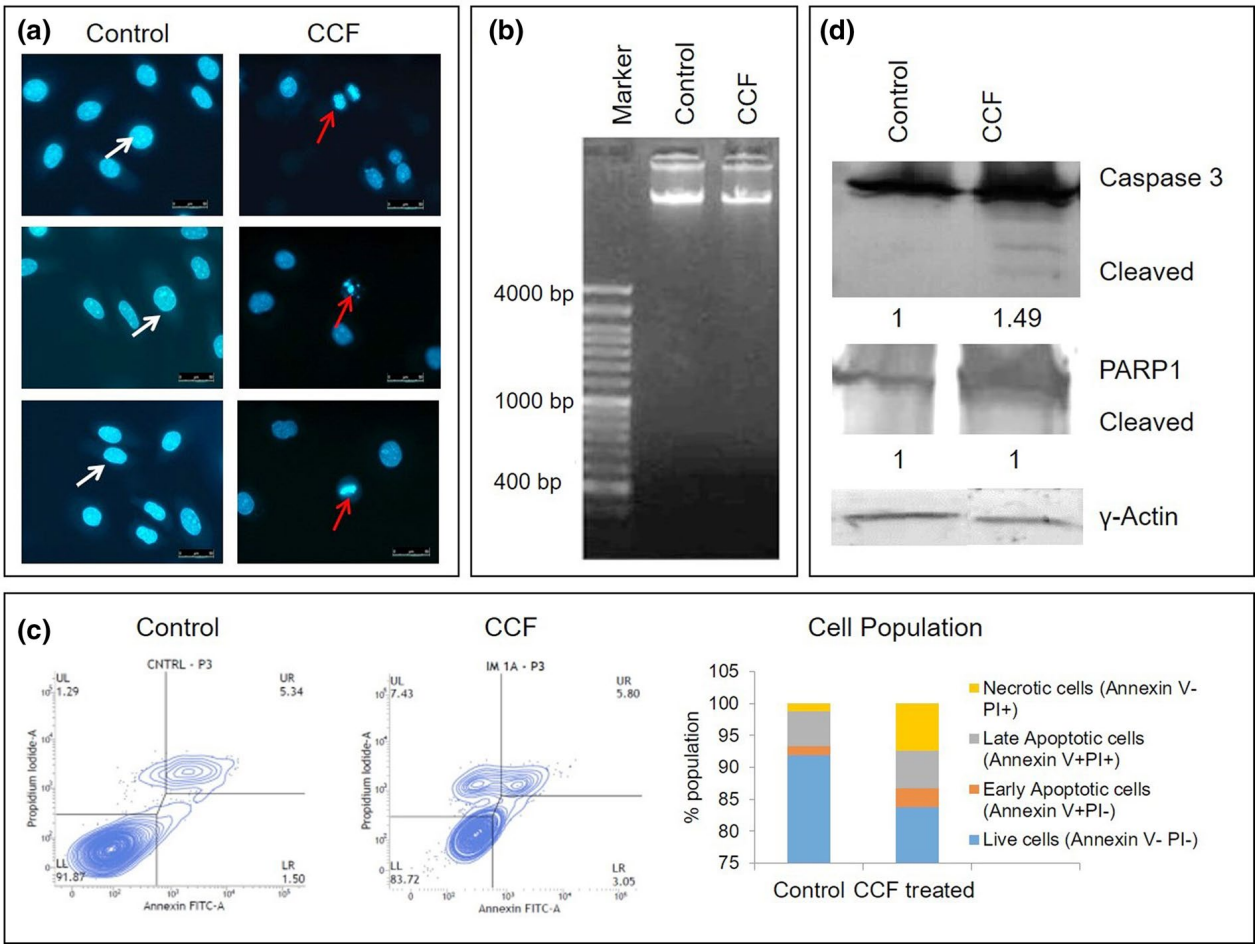
Elucidation of cell death pathway in CCF treated cells, (**a**) Nuclear morphology under fluorescence microscope, scale represents 50 µm, white arrows indicate ovoid shaped normal nuclei and red arrows indicate eroded nuclear morphology under 40 × magnification; (**b**) Extracted genomic DNA run in 1.5% agarose gel showing no DNA laddering; (**c**) Annexin V-FITC/PI assay showing scatter plots with each quadrant representing variable population of cells; LL: viable cells (annexin V − PI-), LR: early apoptotic cells (annexin V + PI-), UR: late apoptotic cells (annexin V + PI +) and UL: dead cells (annexin V − PI +), bar graph showing percentages of different populations of cells; (**d**) Protein expressions for Caspase3 and PARP1, γ-actin served as loading control. Densitometric analysis (ImageJ^1.52A^) showed fold changes of expressions of the proteins were presented below of respective blot pictures. [Blot figures for control and treated sets in (**d**) were cropped from same gel. Detailed information was given as Supplementary Information].

The *nuclear morphology* of treated cells had revealed absence of any apoptotic markers like, condensation and fragmentation of chromatin material (Fig. 3a) in the CCF treated cells under epi-fluorescence microscope. When extracted DNA from both the treated and control sets was run in 1.5% agarose gel, genomic DNA was found in a discrete band in both the control and CCF treated sets. No *DNA laddering* was observed in the treated set (Fig. 3b). Flow cytometric *Annexin-FITC* assay showed no significant increase in apoptotic cells (Fig. 3c). Western blot analysis showed increase (0.5 fold) in cleaved caspase3 (a hallmark apoptotic protein) expression (which was not very significant) along with expression of full length PARP (without any cleaved products) (Fig. 3d). All these observations indicated that in treated set, the cell death pathway was non apoptotic.

### Autophagosome formation

**Figure 4:**
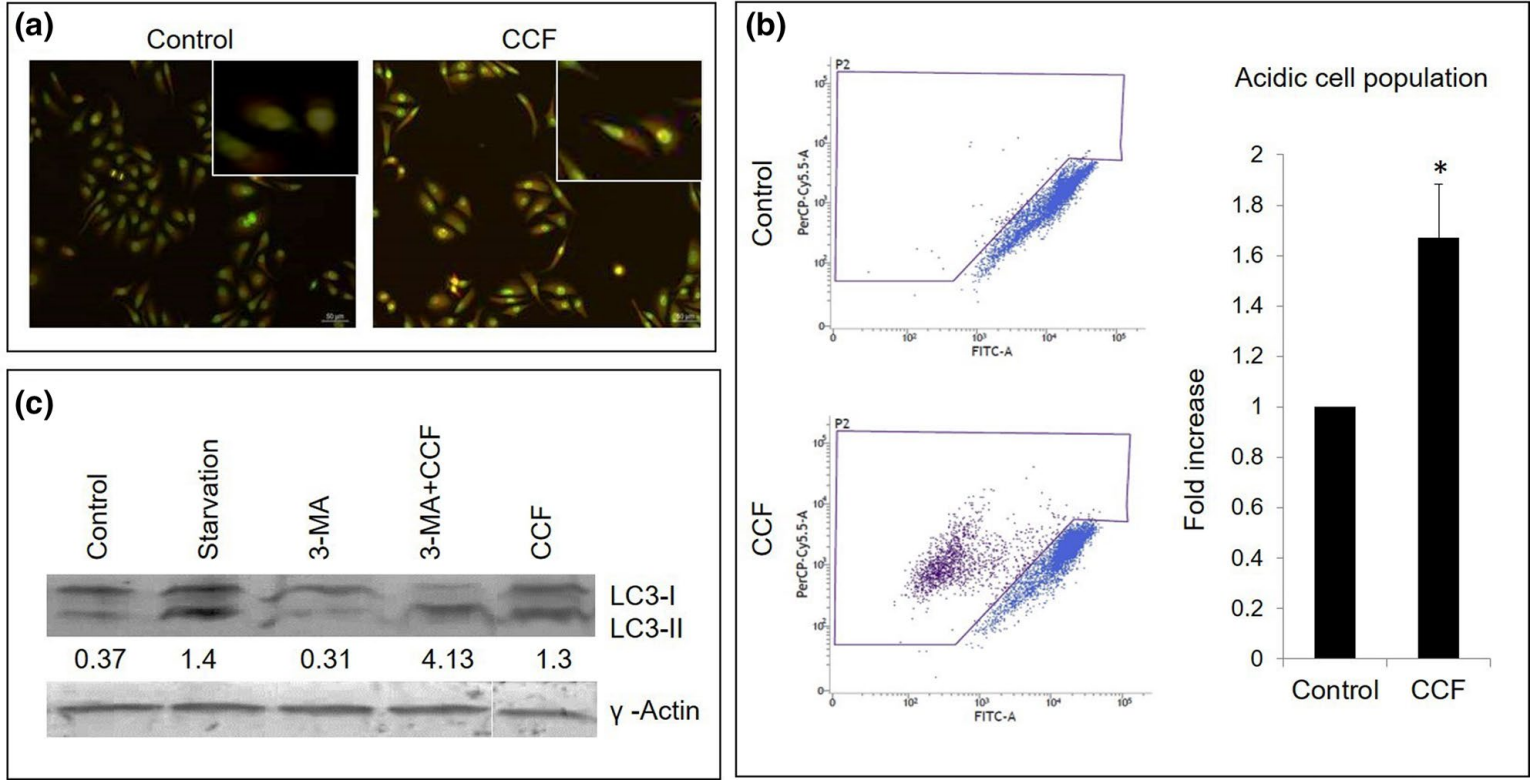
Autophagosome formation in CCF treated cells, (**a**) acridine orange stained cells under fluorescence microscope showing acidic compartments as bright red specs, scale represents 50 µm, inset showing magnified view; (**b**) Scatter plots showing flow cytometric quantification of acidic compartments, bar graph showing percentage of population with acidic vacuoles, values were expressed as mean ± SD of three independent experiments (n = 3); *denotes significant difference between control and treated sets (*P* < 0.05). (**c**) Expressions for autophagic marker protein LC3B, γ-actin served as loading control, densitometric analysis (ImageJ^1.52A^) of LC3B-II/LC3B-I expressions ratios were presented below the respective blot pictures. [Blot figures for different sets in (**c**) were cropped from same gel. Detailed information was given as Supplementary Information].

Staining cells with *acridine orange* revealed autophagosome formation in the CCF treated cells. Epi-fluorescent microscopy showed increase in red fluorescence in the treated cells suggesting acidic vacuole formation (Fig. 4a). Flow cytometric quantification of acridine orange specific cells showed 0.7 fold increase in red fluorescence, confirmed acidic vacuole formation (Fig. 4b). The role of autophagic cell death was more pronounced by increased expression of hallmark autophagic protein LC3B. Conversion of LC3BI to LC3BII act as a marker during autophagosome formation. LC3B-II/LC3B-I expression ratios in treated sets and positive control sets had increased significantly indicating a greater degree of LC3BII lipidation, led towards autophagosomal cargo degradation in CCF treated cells (Fig. 4c).

### Autophagy induction

**Figure 5:**
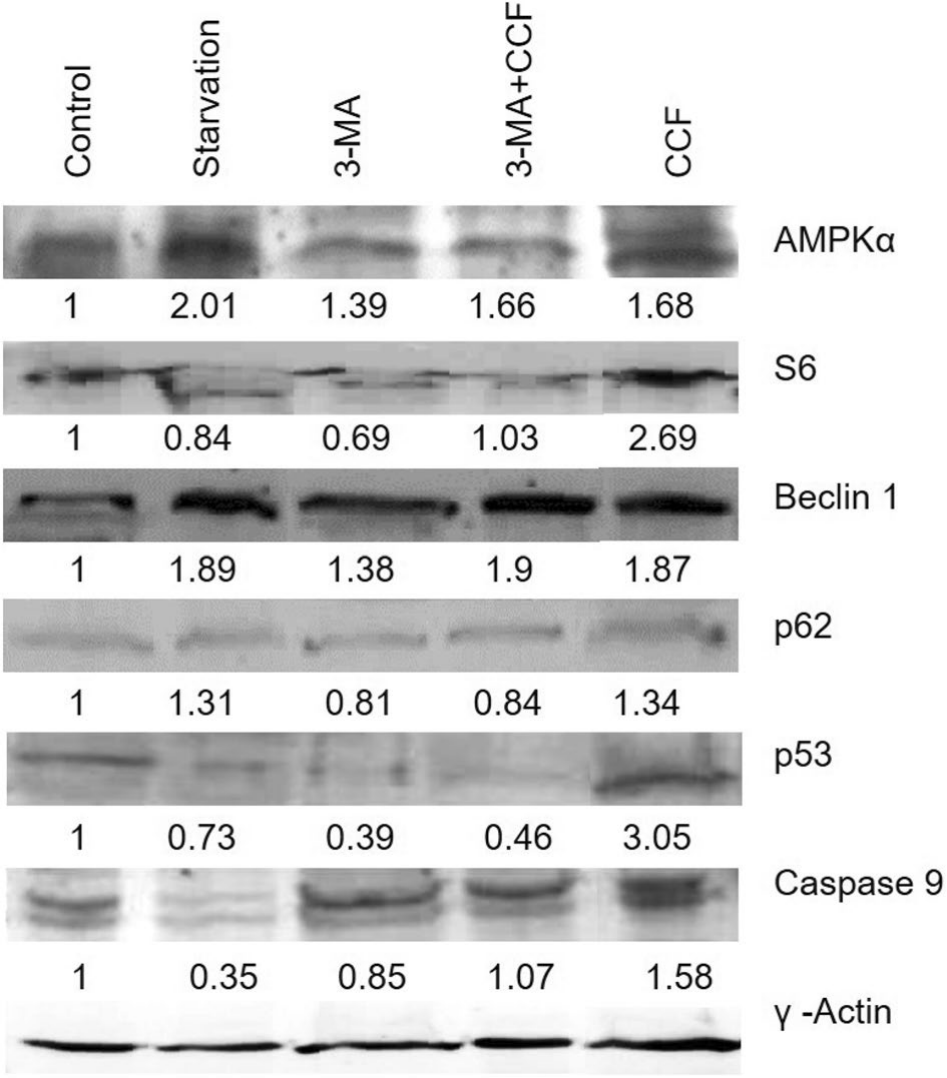
Autaphagy signaling, expressions for autophagic signaling regulator proteins, γ-actin served as loading control. Densitometric (ImageJ^1.52A^) fold changes of expressions of the proteins were presented below the blot pictures. [Blot figures for different sets in were cropped from same gel. Detailed information was given as Supplementary Information].

*Immunoblotting* of key autophagic regulator proteins depicts the autophagic regulation in the CCF treated cells. Expression of AMPKα elevated 0.68 fold in CCF treated set along with 1.01 fold increase in starvation-induced set (positive control). Key autophagic proteins S6, Beclin 1 and p62/SQSTM had increased by 1.69 folds, 0.87 fold and 0.34 fold in the CCF treated sets (Fig. 5).

### In Silico analysis of interaction of compounds present in CCF with autophagic regulator Bcl-2/Beclin 1

**Figure 6:**
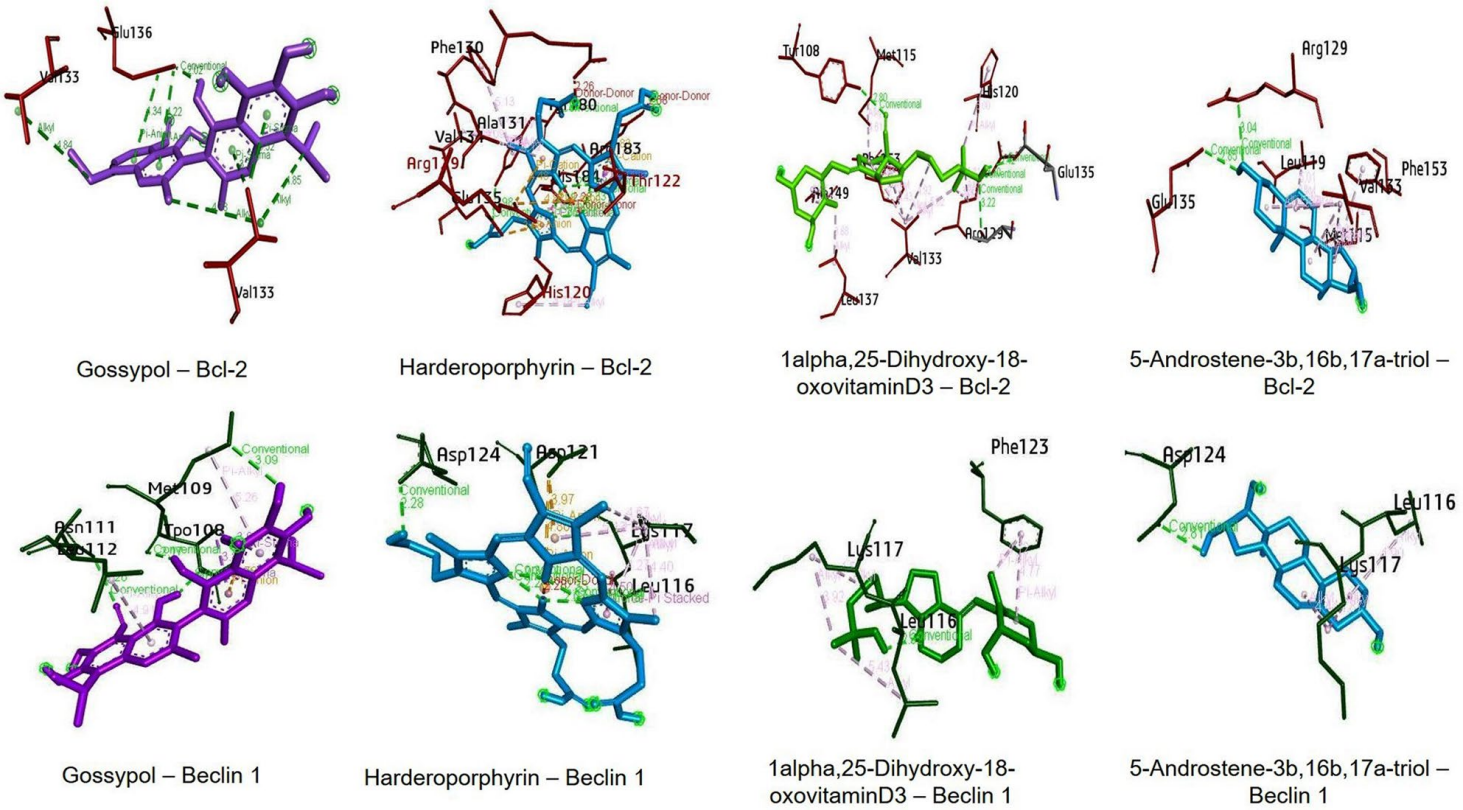
Interaction of three phytochemicals having high banding affinities with Bcl-2 and Beclin1 along with the positive control ligand Gossypol. Color codes for Gossipol; Harderoporphyrin; 1alpha,25-Dihydroxy-18-oxovitaminD3; 5-Androstene-3b,16b,17a-triol were purple, deep sky blue, green and cyan respectively.

Apoptosis is considered as the default pathway of stressed cell, but, failure to induce apoptosis, may lead to autophagy. As autophagic cell death is regulated by Beclin 1 - Bcl-2 interaction, we have tried to study the binding potential of phytochemicals present in the CCF with Bcl-2 and Beclin1. Molecular docking analysis undertaken in the present study revealed that phytochemicals showed high binding affinity towards Bcl-2 and much lower binding affinity towards Beclin1. Out of them, 3 compounds showed more affinity to Bcl-2 (Table 1), compared to Gossypol (positive control). Harderoporphyrin, 1alpha,25-Dihydroxy-18-oxovitamin D3 and 5-Androstene-3b,16b,17a-triol showed − 9.7, − 8.4 and − 8.3 kcal mol^−1^ binding affinity for Bcl-2 while that of Gossypol was − 8.0. Figure 6 showed the amino acid-ligand interactions with the protein targets for the ligands with more binding affinities towards Bcl-2 than Gossypol. Harderoporphyrin was found to bind Bcl-2 via Arg183A (Pi-ion bond), Thr122B (Pi-Sigma bond), His184A (Pi-Pi stacked bond), His120B (Pi-Alkyl bond), Glu135A (Pi-ion bond), Arg129B (2 Pi-ion bonds), Tyr180A (2 H bonds), Ala131A (Alkyl bond), Val134A (Alkyl bond), Phe130A (Alkyl bond), Tyr180A (2 H bonds). While that for 1alpha, 25-Dihydroxy-18-oxovitamin D3 were Arg129B (H bond, 2 Alkyl bonds), Thr132A (H bond), Glu135A (H bond), His120B (H bond), Tyr108B (H bond), His120B (Alkyl bond), Phe153B (Alkyl bond), Val133B (4 Alkyl bonds), Met115B (Alkyl bond), Ala149B (Alkyl bond), Leu137B (Alkyl bond). 5-Androstene-3b,16b,17a-triol interacted with lesser residues and bound via Phe153B (Alkyl bond), Met115B (Alkyl bond), Val133B (4 Alkyl bonds), Arg129B (H bond), Glu135A (H bond), leu119B (Alkyl bond). Interactions of those ligands with Beclin1 was tabulated (Table S2). From these analysis, it was observed that the role of the phytochemicals present in CCF were found to be potent enough to disrupt Bcl-2/Beclin1 interaction and can induce autophagy in the SiHa cells.

**Table 1:** Binding affinities of the phytochemicals present in CCF with Bcl2- and Beclin1.

SI. no	PubChem Id (CID)	Compound name	Binding affinity (Kcal mol^−1^)	
			BCL-2 (PDB if 2XA0)	Beclin 1 (PDB id 6DCN)
1	3081462	Harderoporphyrin	− 9.7	− 5.3
2	52931516	1alpha,25-Dihydroxy-18-oxovitamin D3	− 8.4	− 4.5
3	21252251	5-Androstene-3b,16b,17a-triol	− 8.3	− 5.3
4	3503	Gossypol (control)	− 8.0	− 5.1
5	6536	Triparanol	− 7.5	− 4.8
6	4457488	Clovanediol Diacetate	− 7.4	− 5.0
7	5283077	Pgf 2 alpha isopropyl ester	− 7.0	− 4.3
8	97700	alpha-Phenylcyclohexylglycolic acid	− 6.7	− 4.6
9	5281929	Methyl jasmonate	− 6.2	− 3.9
10	93285	[2-(2-Aminopropoxy)-3-methylphenyl]methanol	− 6.1	− 3.7
11	16757	5-Phenylvaleric acid	− 6.1	− 4.0
12	349300	9-Octadecynoic acid, 12-oxo-	− 6.0	− 3.6
13	5283019	methyl 9,10-epoxy-12,15-octadecadienoate	− 5.8	− 3.5
14	613533	3,4-Bis(methylsulfanyl)quinoline	− 5.7	− 3.4
15	985	Hexadecanoic acid (Palmitic acid)	− 5.6	− 3.1
16	36604	levmetamfetamine	− 5.6	− 3.8
17	5312654	5,9-Octadecadiynoic acid	− 5.5	− 3.5
18	11005	Myristic acid	− 5.3	− 3.3
19	5826	Dextroamphetamine	− 5.3	− 3.5
20	5282733	9-lauroleic acid	− 5.0	− 3.2
21	–	8-keto Eicosane	− 5.0	− 3.0
22	6106	Leucine	− 4.8	− 3.3
23	17083	11-Aminoundecanoic acid	− 4.7	− 3.1
24	67717	Trifluoroacetamide	− 4.5	− 3.0

## Discussion

Autophagy, termed as the Type II programmed cell death, is considered a way to maintain cell homeostasis by recycling cell organelles and macromolecules. This occurs through three steps, first, a double‐membraned structure, termed as autophagosome is formed (during autophagosome formation, Beclin 1 and LC3 proteins play important role), enclosing the nonfunctional organelles and cytoplasmic macromolecules, then, the autophagosome fuses with the lysosome to form autophagolysosome. Then, the lytic enzymes degrade the cargo, enclosed within the vesicles. Amino acids or other compounds, generated from the process are then recycled. Autophagy can be induced as a response to stresses, such as hypoxia, nutrient-starvation or exposure to a cytotoxic agent. So, it’s more like a survival strategy for the stressed cell. But, if the stress is beyond the threshold value, the cells fail to die the canonical apoptotic cell death pathway and opted for autophagy. Autophagy is closely associated with cell proliferation and cancer progression, as impairment of autophagy leads to tumorigenesis, but, on the other hand, autophagy can be used as a therapeutic target for cancer treatment also.

In different types of cancer, an altered expression of autophagic proteins are observed. In liver tumorigenesis, *Beclin 1*, a haplo-insufficient gene has been implicated, similarly, *ATG 5, ATG7* and *p62* are also found to have important tumorigenic role, and suppression of these genes can reduce cancer progression^9^. Reduced Beclin1 expression is also found in several other cancers^10^ and breast cancer^11^.

In cervical cancer also, autophagic proteins show altered expression. In clinical samples of cervical cancer tissues low expression of two autophagic proteins, Beclin 1 and LC3 (microtubule‐associated protein light chain 3) were observed, in comparison to the healthy cervical tissue, which is found to be associated with development of cervical cancers and poor prognosis^12,13^. The result also indicated that, upregulated expression of Beclin 1 are associated with improved survival of cervical cancer cases. Uncontrolled cell proliferation, resistance to apoptosis, recurrences and distal metastasis are the main obstacles in treating cervical cancer^14,15^ may further strengthen the need for regulated autophagy as a therapeutic alternative. Oridonin^16^ (an *ent*-kaurane diterpenoid isolated from *Rabdosia rubenscens*), and Resveratrol^17^ (polyphenol from grape skin) were reported to induce autophagy in cervical cancer cells through autophagosome formation.

In the present study, we tried to explore the potential of *Chaetomorpha brachygona* phytochemicals to induce autophagy in SiHa cells . Most of the algae from marine habitat contain fatty acids as the major phytochemicals^18^. The algal secondary metabolites can interfere with cancer cell receptors and show bioactivity against them. Phytochemicals present in CCF, especially the quinoline derivatives, fatty acids and other major compounds are the prime candidates for the induction of autophagic cell death in cervical cancer cells. Some compounds having structural similarities with the identified compounds were reported to have bioactivities against in vivo and in vitro systems. Molecular docking study revealed the BH3-mimetic role of the phytochemicals detected in CCF, which would had been instrumental in breaking the Bcl-2/ Beclin 1 interaction and induction of autophagy. AutoDock Vina, an open-access program for molecular docking is considered as one of the most validated docking applications. It is well validated against a standard benchmark named as Directory of Useful Decoys by the Watowich group. This program gains a great acceptance in the scientific community due to its ease of access and accuracy^19,20^. In Silico analysis showed high Bcl-2 binding potential of the phytochemicals present in the CCF. Gossypol being a well-known BH3 mimetic^21^ served as the positive control for judging binding affinities of the phytochemicals with Bcl-2. Beclin 1 is the major regulator in apoptosis-autophagy crosstalk. In a default cell death pathway, Bcl-2 binds Beclin 1 by the BH3 domain and inhibits autophagy, but, release of Beclin 1 from the Bcl-2/Beclin 1 heterodimer is a prerequisite for autophagy. It was reported earlier that, BH3 mimetic compounds are able to displace Beclin 1 from the Bcl-2 - Beclin 1 complex by competitive binding and can initiate autophagy^22^. In our study, it is observed that, phytochemicals (23 compounds acted as BH3 mimetics) present in CCF showed more affinity towards Bcl-2, thereby inhibiting its binding to Beclin 1, which help to induce autophagy.

3,4-Bis-(methylthio)-quinoline was found to be one of the major compounds present in the extract. Several quinoline derivatives like chelidonine, nitidine, dictamine were reported as potent anticancer compounds^23–25^. Our docking study revealed strong binding affinity of this compound with Bcl-2 (− 5.7 kcal mol^−1^). GC–MS data clearly showed the abundance of palmitic acid, stearic acid and oleic acid in CCF. Fatty acids like hexadecanoic acid (palmitic acid) and octadecanoic acids (stearic acid) had been reported as anticancer compounds^26^. Palmitic acid (PA), at a concentration of (0.2–0.4 mmol/L), was reported to induce apoptosis in human hepatoma cell line (HepG2) at a dose- and time-dependent manner, Bcl-2 level was found to decrease slightly; whereas, Bax level was found to be elevated markedly^27^. Saturated free fatty acids; such as palmitic acid and stearic acid, can suppress the granulosa cell survival in a time and dose-dependent manner by inducing apoptosis. Western blot analysis showed the down-regulation of Bcl-2, and up-regulation of Bax^28^. NGF diffrentiated-PC12 cells exposed to stearic and palmitic acids, for 24 h, showed loss of cell viability by caspase independent apoptotic cell death with up-regulation of the Fas receptor and ligand^29^. Bioinformatics data showed potential anti-Bcl-2 activity of these fatty acids detected from CCF (Table S1). Methyl jasmonate had found to downregulate HPV E6 and E7 and induced apoptotic cell death in vitro^30^. Effect of methyl jasmonate enhanced when used in combination with cisplatin^31^. It was found to possess high binding affinity (− 6.2 kcal mol^−1^) in our molecular docking study. That study also revealed high binding affinity (− 8.3 kcal mol^−1^) of 5-Androstene-3b,16b,17a-triol towards Bcl-2. One of the androstan derivative also had shown cytotoxicity against human embryonic kidney cells or HEK293 cells at 0.2 µM^32^. Several forms of polyphenolic phenyl valeric acids were found to show antiproliferative activities in different cancer cells^33^. It showed decent binding affinity (− 6.1 kcal mol-^−1^) for Bcl-2. Triparanol (− 7.5 kcal mol^−1^ binding energy for Bcl-2) detected from LC–MS analysis is a cholesterol synthesis inhibitor. It was found to be anti-proliferative and induce apoptosis in multiple human cancer cells by repressing Hedgehog pathway. Triparanol also caused impediment of tumor growth in human lung cancer xenograft model^34^. Vitamin D3 was reported in several literature as autophagy inducer by increasing beclin-1 expression^35,36^. 1alpha,25-Dihydroxy-18-oxovitamin D3 detected in CCF by LC–MS study showed excellent binding affinity (− 8.4 kcal mol^−1^) towards Bcl-2. 8-keto Eicosane is a long chain (C20) alkane, which are considered as natural component of plants. It showed decent binding affinity towards Bcl-2 (− 5.0 kcal mol^−1^).

The bioactive compounds present in the algal extract had not caused cell cycle arrest or inhibited cell cycle progression but induced cell death within the cells. Wound healing assay indicates that the extract possesses cytotoxic and anti-proliferative properties to the SiHa cells as no directional cell migration was observed. Some of the active phytochemicals present in the extract might have triggered the death pathway in the treated cells. No condensation of nuclear morphology and absence of DNA laddering assay suggested a different cell death mechanism than apoptosis. However, MMP assay result of treated set showed mitochondrial membrane disruption along with expression of cleaved caspase3. Decreased ATP production in those cells might had initiated AMPK dependent autophagic cell death pathway.

Increased intracellular ROS activates ATM, which phosphorylates LKB1 protein at Thr366, in turn activates AMPK dependent pathway. AMPK upregulates TSC proteins leading towards cell death (including autophagy) by blocking mTORC1 proteins. It is evident that under stressed condition damaged mitochondria release cytochrome c which in turn activates caspase dependent pathways. Flow cytometric analysis with Rh123 had shown decreased mitochondrial membrane potential with ROS accumulation in treated SiHa cells. This indicates that, internal stress induced due to algal compounds may lead to AMPK activation**.** A study on yeast had indicated the regulatory activity of mitochondria on autophagic death, where the authors had shown that the change in MMP as a regulator of autophagic flux rather than ATP production^37^. It was also reported that depolarization of a limited subset of mitochondria might results in selective autophagosomal elimination of them^38^.

Microscopic analysis had revealed the formation of autophagic bodies appeared as diffused red specs and quantitatively validated by flow cytometric analysis. In treated sets and positive control (starved sets), the expression of hallmark autophagic protein LC3-II was found to be elevated significantly along with expressions of S6, Beclin1, p62/SQSTM1. Down regulation of Bcl-2 expression (Fig. S3) was in accordance with the increased Beclin 1and autophagy induction. In autophagic cells, p53 activates p62 for the autophagy induction and plays a vital role for inhibition of anti-autophagic protein mTOR1 as well as initiates AMPKα dependent cascade in cells. TSC1 and TSC2 are two key regulator for initiation of AMPKα dependent pathway by decreasing Rheb protein resulting downregulation of mTORC1. While S6 protein downregulates pULK1 at upstream cascade of autophagy. In both the treated and starved sets, the expression of autophagic hallmark protein LC3BII was found to be increased significantly. At maturation the role of this protein for autophagosome formation and fusion of autophagic vesicles is crucial. Finally, the detrimental process of autophagy was mediated by self-degradation of the autophagosomal cargo. Atg 5–12 and p62 is responsible for LC3BII maturation in cells. Increased expression of Atg 5–12 in CCF treated cells is indicative of this (Fig. S3).

It was observed that the expressions of some apoptotic hallmark proteins like Caspase 3, PARP1 were not increased significantly in case of the treated cells. These results were in accordance with no fragmentation of DNA and less Annexin V positive cells. All these observations obtained from immunoblotting point towards autophagic death in CCF treated cells.

**Figure 7:**
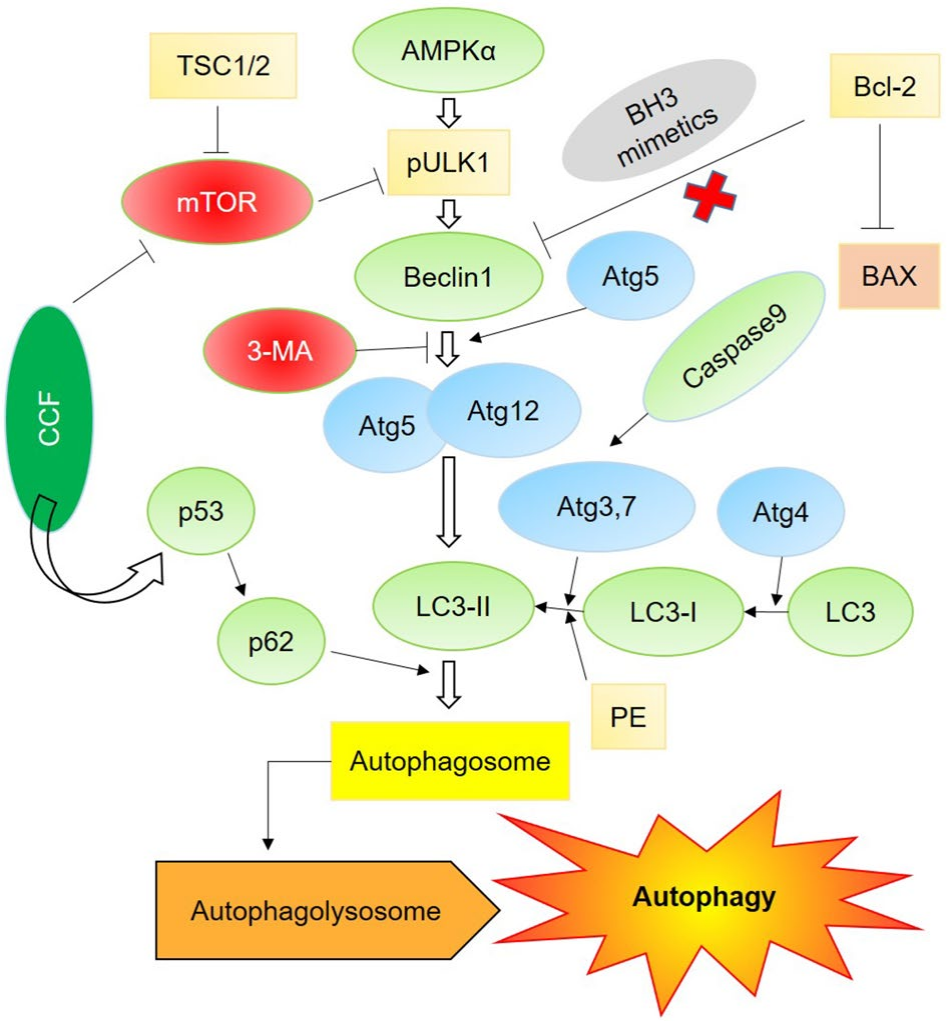
Proposed mode of action of the CCF on the SiHa cells inducing autophagy.

From the experimental findings, it can be observed that, several bioactive compounds are present in the algal extracts, all these compounds are not able to induce cell death independently, however, it is the synergistic effect of the metabolites, which are responsible for the increased level of intracellular ROS which might have induced Beclin1 dependent autophagic cell death in treated sets (Fig. 7).

As the study was limited to only one type of cervical cancer cells, it will be premature to comment that, it will effectively inhibit other types of cancer cells also, but, as the green alga is rich in unsaturated fatty acids and novel phytochemicals, undoubtedly it can be utilized for its nutraceutical values. Compounds with high binding affinity to Bcl-2, detected/identified by molecular docking analysis are of main interest, future research can utilize these molecules for therapeutic purposes.

## Material and methods

### Collection of algal material

*Chaetomorpha brachygona* was collected from Sandeshkhali, West Bengal, India (22°21·471´north & 88°52·792´ east) of Indian Sundarbans. For identification, the collected algal specimens was preserved in FAA (formaldehyde: acetic acid: alcohol) solution and are maintained with proper accession numbers at herbarium of University of Calcutta (CUH).

### Extract preparation

Algal samples were thoroughly washed with water and air dried. After drying the algae were cut into small pieces and extracted with about five times methanol at dark for two nights. The methanolic extract was concentrated and defatted with petroleum ether (Merck) and mixed with distilled water (7:3). Resulting mixture was fractioned with chloroform (Merck) to get the *Chaetomorpha* chloroform fraction (CCF). CCF was lyophilized and dissolved in dimethyl sulfoxide (DMSO) at a concentration of 100 μg/μl for further use.

### Characterization techniques

*GC–MS* Lyophilized CCF was dissolved in GC grade dichloromethane (Merck) and derivatized with BSTFA (Sigma) following manufacturer’s protocol and run in an Agilent HP5 column (30 × 0.25x0.2) in temperature gradient of 70–260 °C with a ramping rate of 5 °C/min and helium flow of 1 ml/min in a GC/MS system (Agilent 7890A-5975C). Detection and identification of the isolated phytochemicals was done by NIST 2011 library.

*LC–MS* Lyophilized CCF was dissolved in HPLC grade methanol (Merck) and 3 µl of extract was injected in an Agilent G1316C column and run for 30 min with water and acetonitrile as mobile phase. The system is interfaced to a TOF/ Q-TOF mass spectrometer with an ion source analyzer- Dual AJSESI. The analysis was done from IIT, Mumbai.

*Column chromatography* A slurry was made from concentrated CCF and loaded in a glass column having silica gel (60–120). Elution was started with petroleum ether and then increasing concentrations of ethyl acetate and later with methanol. Sub-fractions were collected, concentrated and pooled. Pure fractions were tested for their cytotoxicity by MTT assay. Cytotoxic fractions were identified by NMR, ESI–MS studies.

### Cell culture and treatment

Cervical cancer cell line (SiHa) was obtained from NCCS, Pune and maintained in Eagle’s minimal essential medium (MEM) supplemented with 10% Fetal Bovine Serum (Gibco) and 1% antibiotic–antimycotic solution (Penicillin, Streptomycin and Amphotericin in 0.9% normal saline) at 37 °C in a humidified incubator having 5% CO_2_.

IC50dose of CCF was used to treat the cells for 24 h and various tests were performed.

### Cell cycle analysis

After treatment, cells were washed with chilled PBS and fixed using 70% ethanol (Merck). After RNase (Thermo Scientific) treatment, total DNA was stained with propidium iodide (Sigma) and flow cytometric analysis was carried out.

### Apoptosis assay (Annexin V-FITC/PI double staining)

After treatment, cells were harvested and washed with PBS. Then according to manufacturer’s protocol (BD 556,547) cell were stained with FITC conjugated Annexin V and Propidium Iodide for 15 min at dark and subjected to flow cytometry (BD FACSVerse).

### Intracellular ROS detection assay

TBHP (2 h) and NAC (30 min) were used as positive control and ROS inhibitor. After treatment, cells were harvested and washed with PBS and stained with DCF-DA (ABCAM) for 15 min at room temperature. DCF-DA stained cells were quantified flow cytometrically (BD FACSVerse) with an excitation and emission wavelength of 495 nm and 529 nm respectively.

### Loss of mitochondrial membrane potential (MMP)

After treatment, cells were harvested and washed with PBS. After incubation with Rh 123 (10 μg/ml) for 10 min depolarized cell population was measured flow cytometrically (BD FACSVerse) at 507 nm excitation and 529 nm emission. Flowlogic software was used to analyze the data.

### Wound healing assay

Monolayer SiHa cells were grown in dish for overnight. A scratch was made at the centre of the culture dish with a micro pipette tip in one direction. Treatments were added accordingly and incubated for 48 h. Directional migrations of the treated cells were compared with that of control sets and photographed under phase contrast microscope.

### Nuclear morphology

Cells were cultured on pre-coated poly-lysine cover glasses. After treatment, cells were washed with PBS followed by fixation in 70% ethanol. Fixed cells were stained with Hoechst 33,258 (2 μg/ml) (Sigma) in dark for 5 min. Stained cells were mounted in PBS containing 10% glycerol (Merck), 2% N-propyl gallate (Sigma) and visualized under epi-fluorescence microscope (Olympus).

### DNA fragmentation assay

After treatment, cells were harvested and total DNA was extracted and separated by agarose gel electrophoresis after staining DNA with Ethidium bromide and documented under gel documentation system (UVP Multidoc-It).

### Study of acidic vacuoles

For microscopic analysis, cells were grown on poly-lysine coated cover glasses. After treatment, 1 μg/ml of acridine orange (Sigma) was added for 5 min after washing the cover slips with PBS and cells were visualized under epi-fluorescence microscope (Olympus). For flow cytometric (BD FACSVerse; 550 nm excitation, 600 nm emissions) analysis, 1 μg/ml of AO was added to the trypsinized cell suspensions for 10 min. Flowlogic software was used to analyze the FACS data.

### Western blot analysis

After treatment, total protein was isolated from the cells and separated in 10% polyacrylamide gels, and transferred to nitrocellulose membrane (Pall). A positive control set for autophagy was induced by serum starvation for 3 h following the protocol from Zhang et. al. 2016. After transfer, membranes were incubated with primary antibodies [p53, AMPK, Caspase 3, Caspase 9, PARP1 (1:500); p62, S6, Beclin1 (1:750); LC3B, γ-actin (1:10,000)] for overnight at 4 °C. After washing, alkaline phosphatase (AP) conjugated secondary antibodies were added and incubated for 2 h. NBT-BCIP (Sigma) was used as substrate to develop the bands. Results were photographed by gel documentation system (UVP Multidoc-It) and densitometric analysis was done by NIH-ImageJ^1.52A^ software.

### In Silico molecular docking analysis

#### Preparation of ligands

Twenty-two (22) phytochemicals, as determined by GC–MS and LC–MS were considered as ligands for the molecular docking study against target protein Bcl-2 and Beclin1. Known inhibitor of Bcl-2, Gossypol (CID 3503), was used as control ligand against target proteins. The three-dimensional structures of these compounds were downloaded from the PubChem database^39^ in .SDF format. Ligand energy optimization and conversion in .PDB format was done by Avogadro software before conducting the molecular docking analysis.

#### Preparation of target proteins

Three-dimensional crystal structures of the target proteins namely apoptosis regulator Bcl-2 (PDB id 2XA0, Chain A and B, 2.70 Å, X-ray diffraction) and Beclin1 BH3 domain (PDB id 6DCN, Chain C, 2.44 Å, X-Ray Diffraction) were downloaded from RCSB PDB database^40^. Structural optimization of the protein targets was performed by UCSF Chimera software^41^ & AutoDock tools^19^ and the proteins were later saved in PDBQT format. Active sites of the proteins were predicted by combining the CASTp 3.0 server^42^ and UCSF-Chimera.

#### Molecular docking

Molecular Docking analysis was performed by AutoDock Vina 1.12^19^ for evaluating their inter-atomic interactions and their binding affinities (Kcal mol^−1^) after preparation of ligands and receptors. After the minimization process, the grid box resolution was set along the x, y, and z points (size and center), respectively in the predicted active sites. For the protein Bcl-2 (PDB id 2XA0) and Beclin1 (PDB id 6DCN) the grid box coordinates were set as 55.967, − 18.930 & − 28.753 and 88, 66 & 60 (x, y, z center and size) and 25.527, 27.618 & 29.308 and 60, 44 & 84 (x, y, z center and size) respectively. Each of the ligands, including the control Gossypol were docked with both the protein targets. The docked structures were visualized in PyMOL (Schrödinger)^43^, and the interaction analysis of amino acid residues and ligands were performed by BIOVIA Discovery Studio^44^ 2020 software.

### Statistical analysis

All data were plotted as average with standard deviations. Significance of the quantitative data was analyzed by Student’s T-Test (*P* ≤ 0.05).

## Supplementary information

Supplementary Information.

## Data Availability

All data generated or analyzed during this study are available.
