# Supplementary material for: Chloroform fraction of Chaetomorpha brachygona, a marine green alga from Indian Sundarbans inducing autophagy in cervical cancer cells in vitro
Source: Sci Rep. 2020 Dec 11;10:21784. doi: 10.1038/s41598-020-78592-9 (PMC7733477; doi:10.1038/s41598-020-78592-9)

Supplementary Information

Title: Chloroform fraction of *Chaetomorpha brachygona,* a marine green alga from Indian Sundarbans inducing autophagy in cervical cancer cells *in vitro*

Indira Majumder[1], Subhabrata Paul[2], Anish Nag[3], Rita Kundu[1]*

[1] Department of Botany, University of Calcutta. 35, Ballygunge Circular Road, Kolkata- 700019

[2] School of Biotechnology, Presidency University, Canal Bank Rd, DG Block, Action Area 1D, New Town, West Bengal 700156

[3] Department of Life Sciences, CHRIST (Deemed to be University), Bangalore, 560029, India

* rkbot@caluniv.ac.in

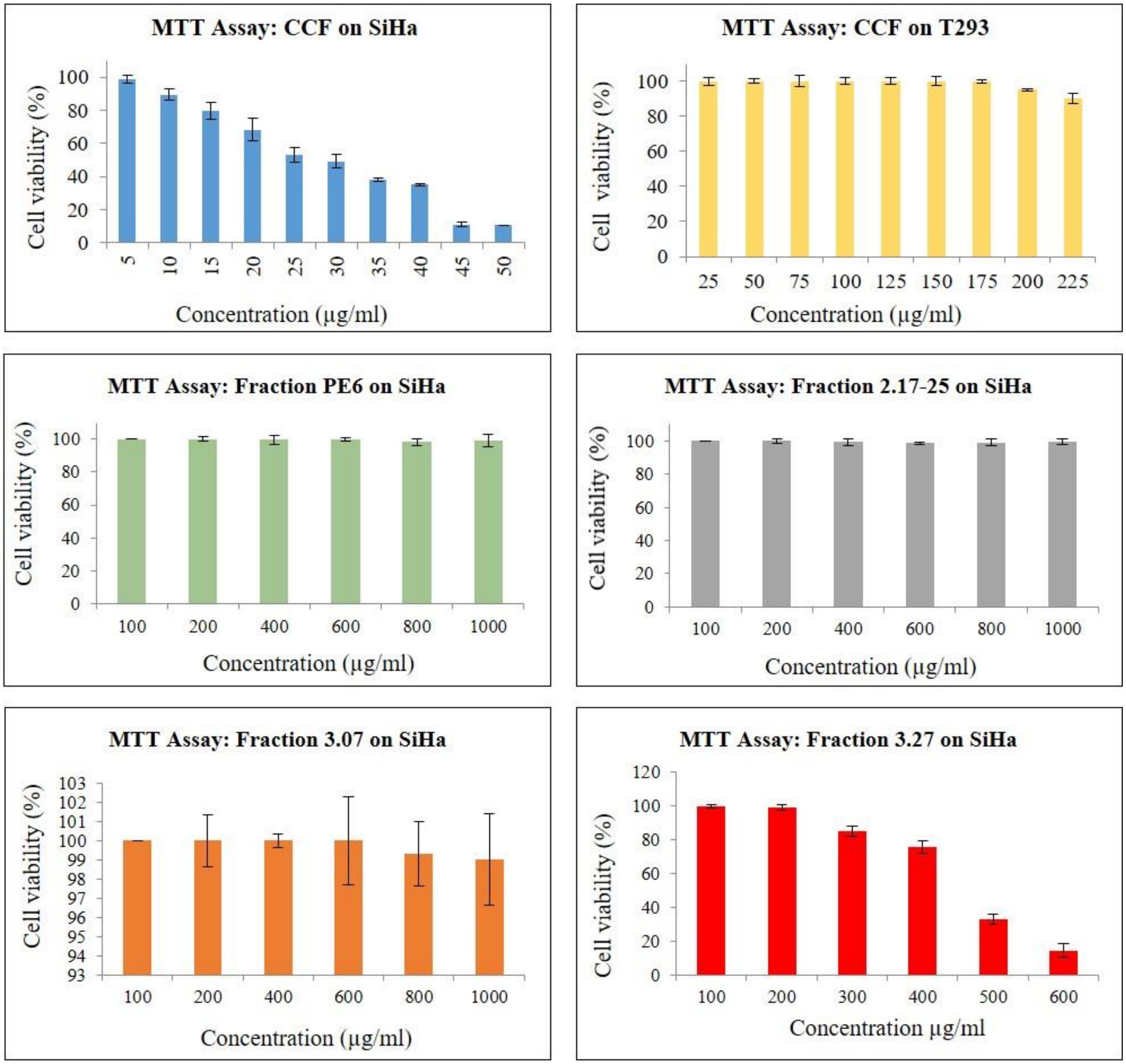

Fig S1 Cell viability of CCF on SiHa and 293T cells along with that of the isolated fractions on SiHa cells.

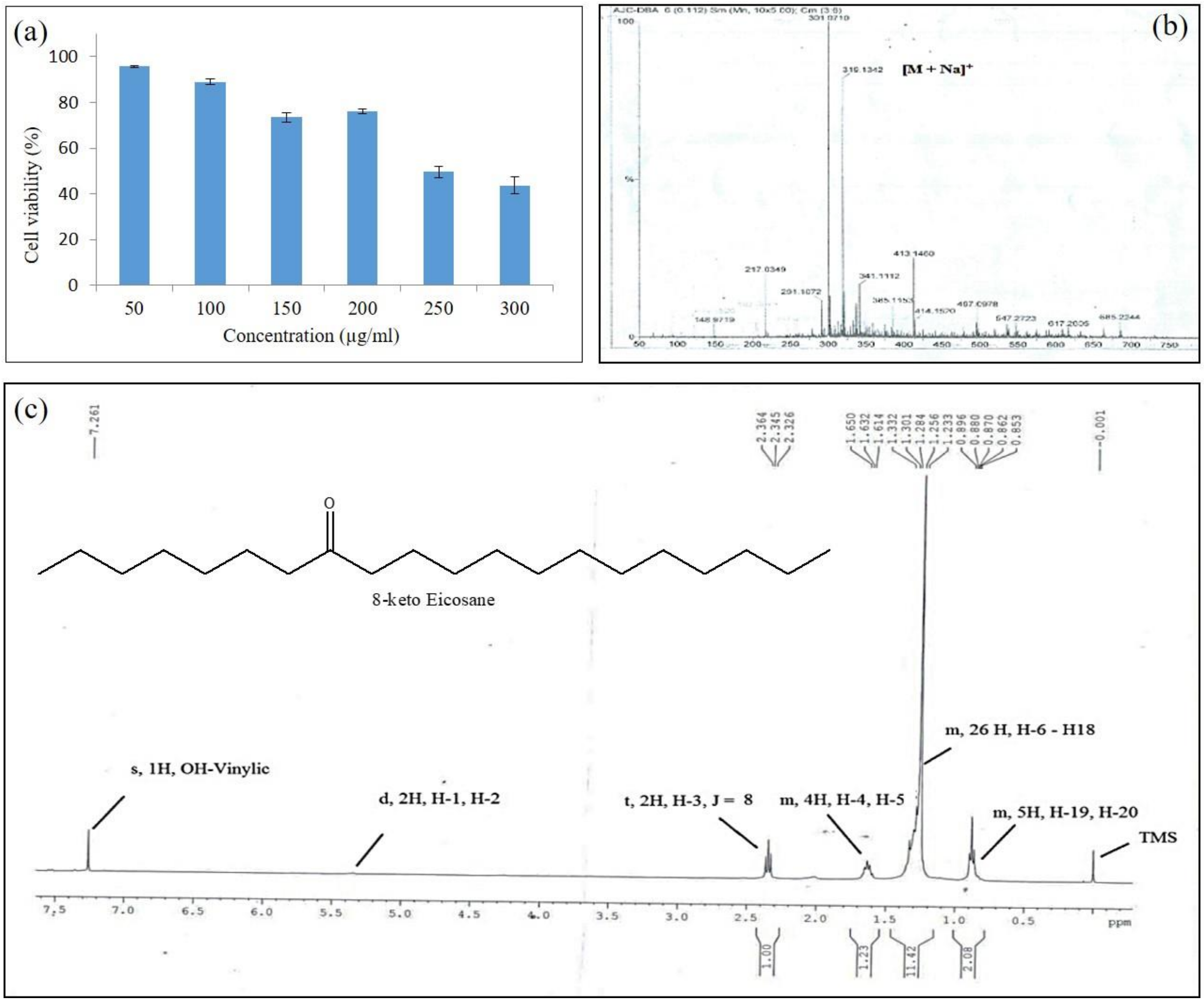

Fig S2 Structural elucidation and cytotoxicity of fraction Fr. 10.1. (a) MTT assay on SiHa cells; (b) ESI-MS; (c) 1H NMR

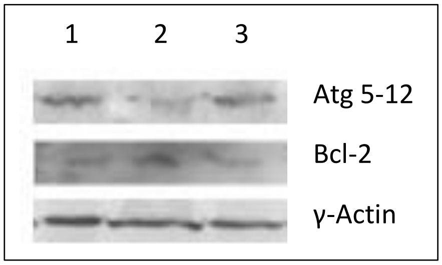

Fig S3 Western blot of autophagic regulators in SiHa cells. Lane 1: control, lane 2: positive control (starvation), lane 3: CCF treated cells.

Table S1. Phytochemicals detected by GC-MS and LC-MS. *denotes compounds showing BH3 mimicking activity as determined by *in silico* analysis.

	GC-MS		LC-MS	
Sl. No.	Name of Compounds	Retention Time (min)	Name of Compounds	Retention Time (min)
1	N,N'-Bis (trimethylsilyl) trifluoroacetamidine*	4.989	Leucine*	0.98
2	Tris(trimethylsilyl)borate	5.645	Triparanol*	0.989
3	Tris(trimethylsilyl)carbamate	6.465	11-amino-undecanoic acid*	1.02
4	Androstan-3-one, 17-hydroxy-4,4-di-methyl-, (5.alpha.,17.beta.)	6.753	Methyl jasmonate*	5.655
5	3,4-Bis-(methylthio)-quinoline*	7.559	Benzenemethanol, 2 (2-aminopropoxy)-3-methyl-*	7.159
6	1-Propanone, 1,3-diphenyl-3-(trimethylsilyl)-	9.874	5-Phenylvaleric acid*	7.159
7	Silanol, trimethyl-, phosphate (3: 1)	12.276	alpha-Phenylcyclohexylglycolic acid*	9.45
8	5-Nitro-3-phenyl-1H-indazole	24.186	Dihydrodeoxystreptomycin	10.064
9	Tetradecanoic acid, trimethylsilyl ester*	25.343	5-Androstene- 3b,16b,17a-triol*	11.762
10	Hexadecanoic acid, trimethylsilyl*	29.134	Pro Arg Asp	12.993
11	3,6-Dioxa-2,4,5,7-tetrasilaoctane, 2,2,4,4,5,5,7,7-octamethyl-	31.961	1alpha,25-dihydroxy-18-oxovitamin D_3*	14.369
12	Octadecanoic acid, trimethylsilyl ester*	32.918	Clovanediol Diacetate*	15.08
13			9-lauroleic acid*	15.081

14	5,9-octadecadiynoic acid*	15.457
15	12-oxo-9-octadecynoic acid*	15.461
16	PGF2alpha isopropyl ester*	16.524
17	methyl 9,10-epoxy-12,15-octadecadienoate*	17.828
18	Harderoporphyrin*	18.261
19	Dodecaprenyl phosphate-galacturonic acid	18.661
20	Levmetamfetamine*	26.417
21	Dextroamphetamine*	26.445

Table S2. Interaction sites of selected phytochemicals with Bcl-2 and Beclin 1.

Protein (PDB id)	Ligand (PubChem id)	Interaction Sites
BCL2 (2XA0)	Gossypol (CID3503)	Glu136A (2 Pi-Anion bonds, H bond),Val133A (Alkyl bond),Val133B (2 Pi-Sigma bonds, 2 Alkyl bonds)
	Harderoporphyrin (CID3081462)	Arg183A (Pi-ion bond),Thr122B (Pi-Sigma bond),His184A (Pi-Pi stacked bond), His120B (Pi-Alkyl bond),Glu135A (Pi-ion bond), Arg129B (2 Pi-ion bonds), Tyr180A (2 H bonds), Ala131A (Alkyl bond), Val134A (Alkyl bond), Phe130A (Alkyl bond),Tyr180A (2 H bonds)
	1alpha,25-Dihydroxy-18-oxovitamin D3 (CID52931516)	Arg129B (H bond, 2 Alkyl bonds), Thr132A (H bond), Glu135A (H bond), His120B (H bond), Tyr108B (H bond), His120B (Alkyl bond), Phe153B (Alkyl bond),Val133B (4 Alkyl bonds), Met115B (Alkyl bond), Ala149B (Alkyl bond), Leu137B (Alkyl bond)
	5-Androstene-3b,16b,17a-triol (CID21252251)	Phe153B (Alkyl bond),Met115B (Alkyl bond),Val133B (4 Alkyl bonds),Arg129B (H bond), Glu135A (H bond),leu119B (Alkyl bond)
Beclin 1 (6DCN)	Gossypol (CID3503)	Asn111C (H bond), Tpo108C (2 H , Pi-Anion and Pi-Sigma bonds), Leu112C (Pi-Alkyl bond), Met109C (H and Pi-Alkyl bonds)
	Harderoporphyrin (CID3081462)	Leu116C (Amide-Pi stacked bond), Lys117C (4 Pi-Alkyl bonds), Asp121C (Pi-Anion bond), Asp124C (H bond)
	1alpha,25-Dihydroxy-18-oxovitamin D3 (CID52931516)	Leu116C (Pi-Alkyl bond), Lys117C (2 Alkyl bonds), Phe123C (2 Alkyl bonds)
	5-Androstene-3b,16b,17a-triol (CID21252251)	Leu116C (Alkyl bond), Lys117C (2 Alkyl bonds), Asp124C (H bond)

Additional gel pictures

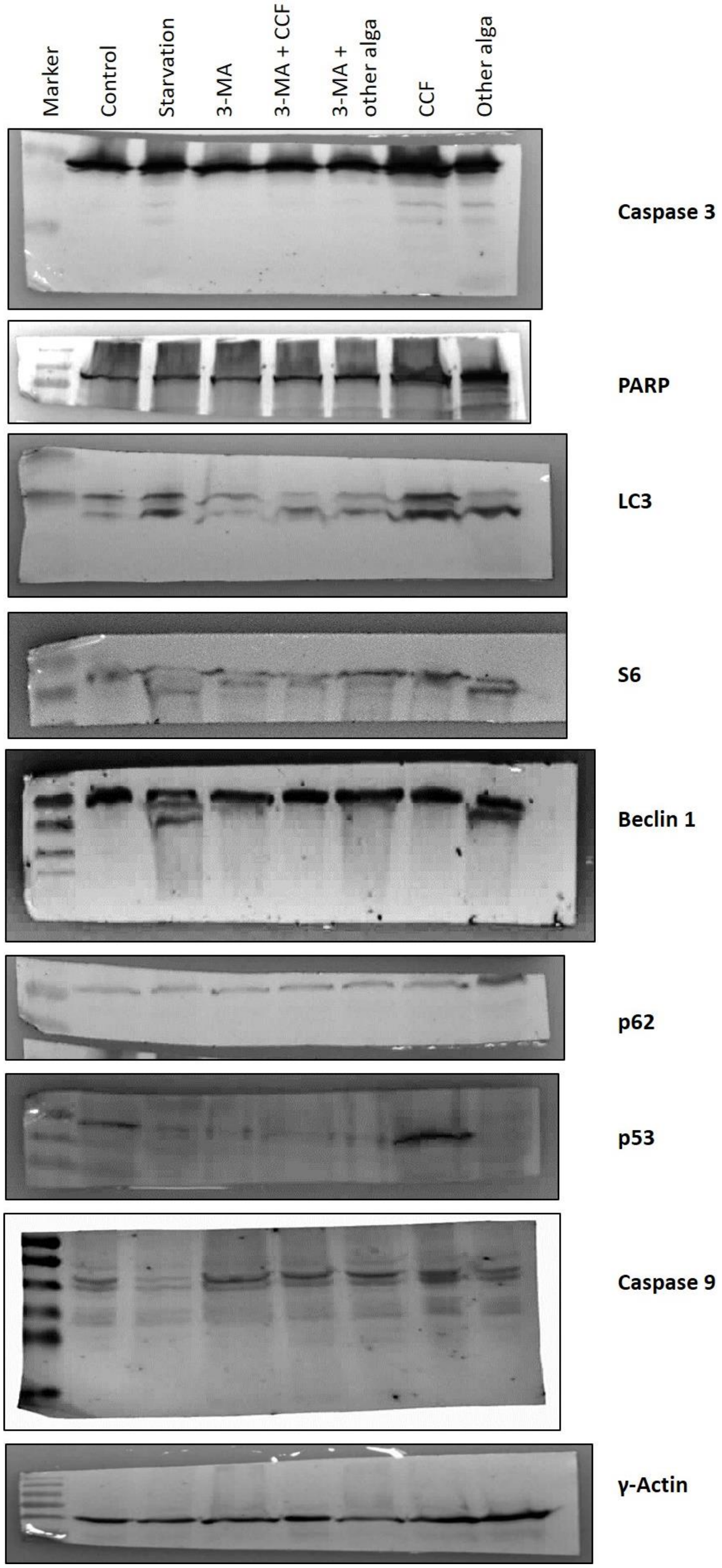

Supplement: Supplementary file 1 — Supplementary Information. [file 41598_2020_78592_MOESM1_ESM.pdf]
